# Supplementary material for: Charged Residue Implantation Improves the Affinity of a Cross-Reactive Dengue Virus Antibody
Source: Int J Mol Sci. 2022 Apr 11;23(8):4197. doi: 10.3390/ijms23084197 (PMC9027083; doi:10.3390/ijms23084197)
Supplement: Supplementary file 1 [file ijms-23-04197-s001.zip › ijms-1640484-supplementary.pdf]

Figure S1. RMSD values for all MD simulation runs.

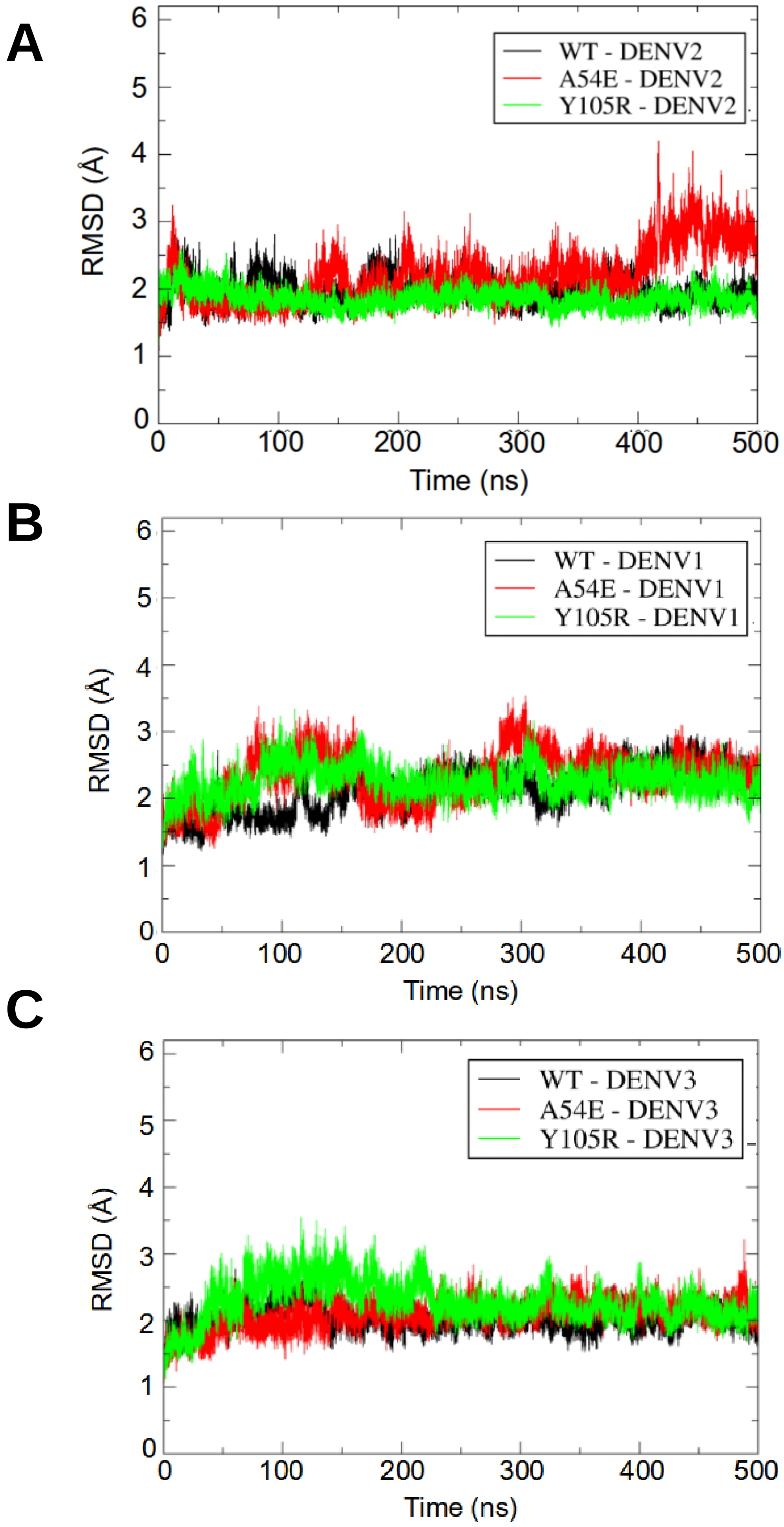

Figure S2. Energy contribution of residues that interacted with Lys325 in the Y105R.H – DENV3 EDIII complex. The plot corresponds to the heatmap in Figure 8A . The bars represent averages and solid lines represent the standard deviation. The EDIII, 1A1D-2 heavy chain and light chain are designated as A, H and L chain, respectively.

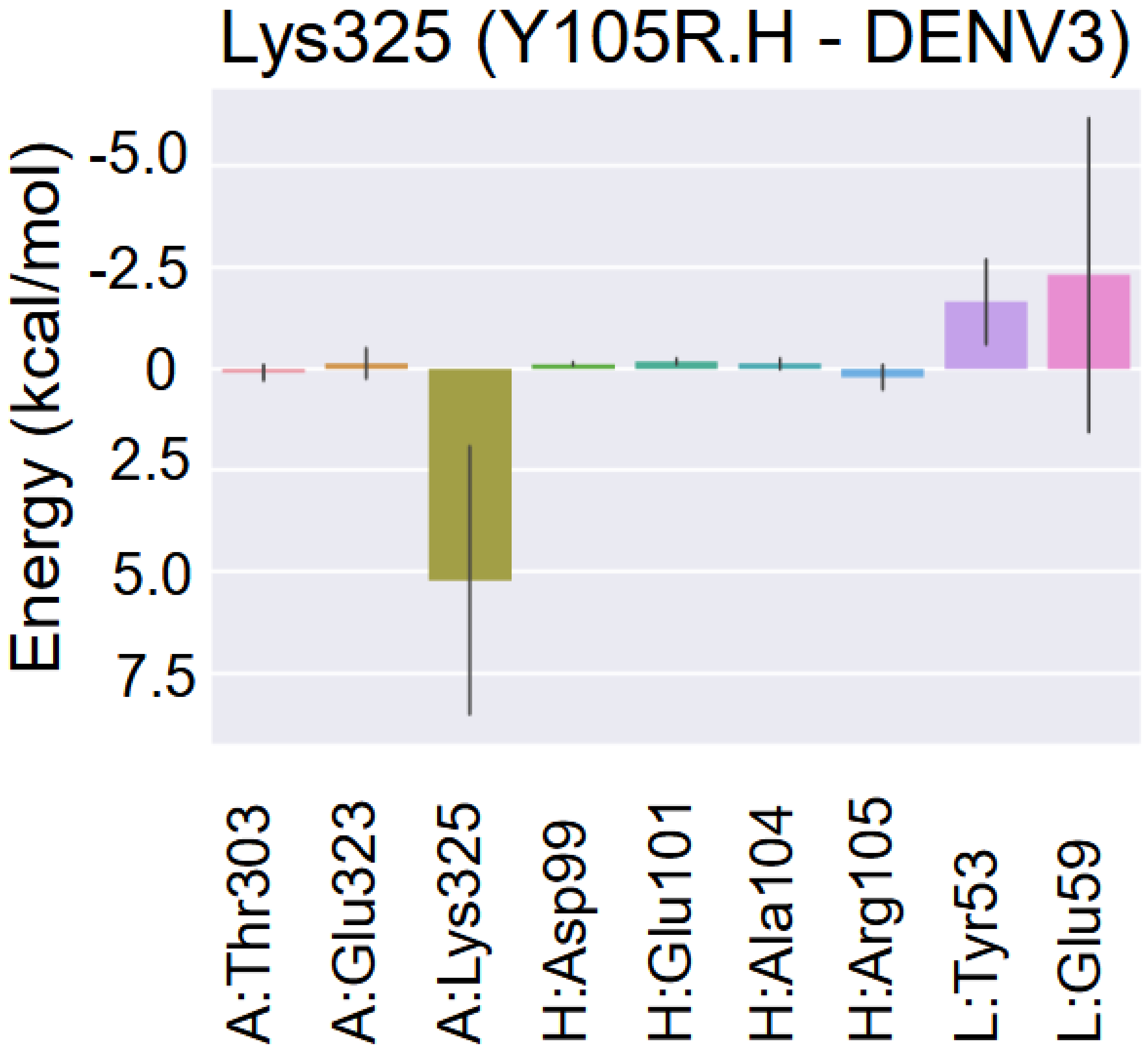

Figure S3. MD simulation of Y105R.H with DENV1 (0 to 500-ns). (A) Energy decomposition analysis: heat maps show energy contribution of residues that interact with Arg105.H per frame in the Y105R.H – DENV1 EDIII complex. Twenty frames with a 25-ns interval were extracted from the full simulation. Red arrows highlight residues strongly interacting with Arg105.H. **(B)** Energy contribution of residues that interacted with Arg105.H, corresponding to the heatmap in (A). The bars represent averages and solid lines represent the standard deviation. The EDIII, 1A1D-2 heavy chain and light chain are designated as A, H and L chain, respectively.

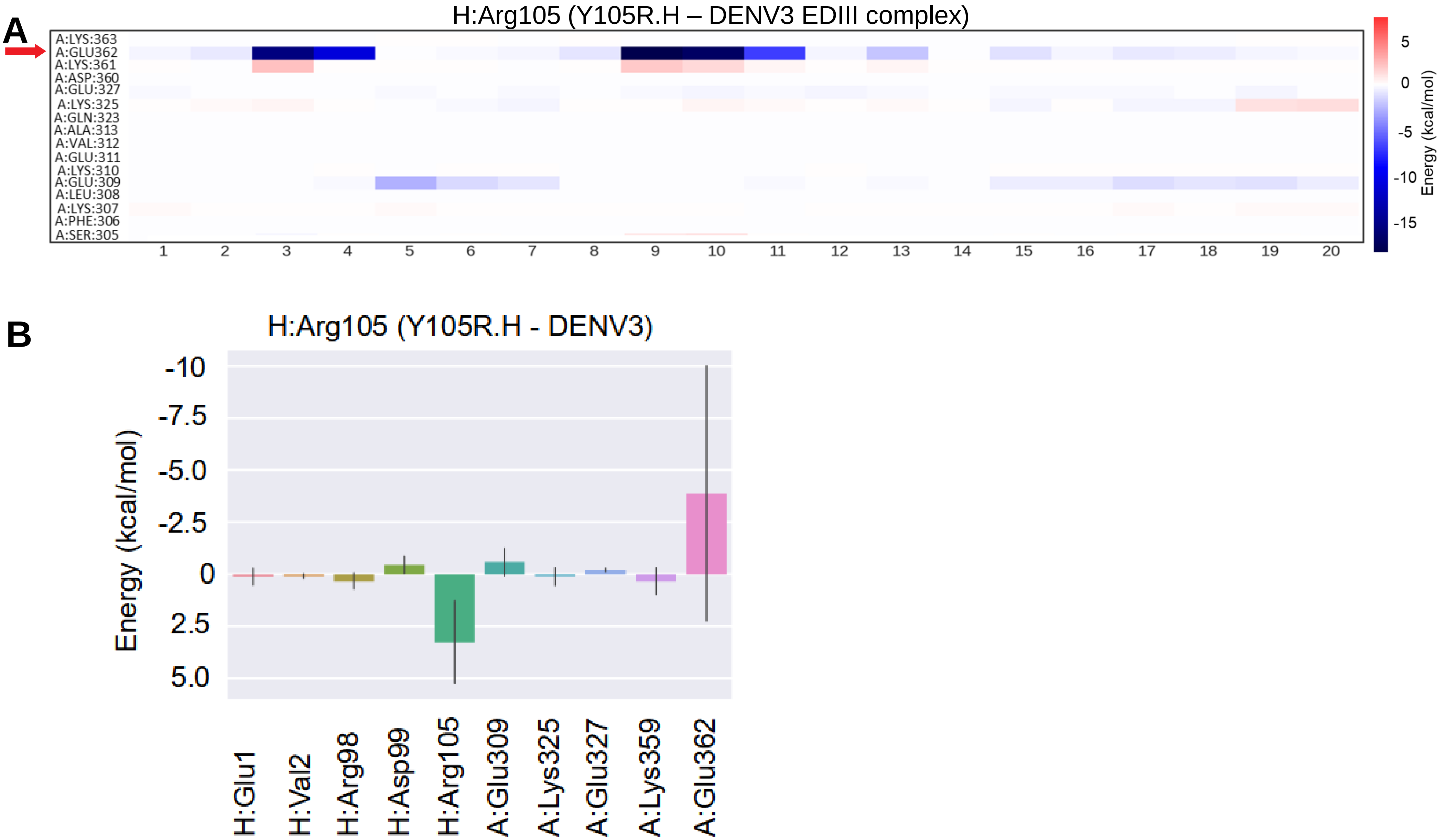

Table S1. List of primer pairs used for cloning and mutagenesis.

**Primer Names**	**Sequence（5′→3′）**
**Cloning**	
**pMal-c2X-DV1-F**	GCAGACTAATTCGAGCTCCATGGGTATGAGCTAC
**pMal-c2X-DV1-R**	TGCCTGCAGGTCGACTCTAGATTATTAGCCTTTTTTGAACCA
**pMal-c2X-DV2-F**	GCAGACTAATTCGAGCTCCAAAGGCATGTCTTAC
**pMal-c2X-DV2-R**	TGCCTGCAGGTCGACTCTAGATTATTAACCTTTTTTGAACCA
**pMal-c2X-DV3-F**	GCAGACTAATTCGAGCTCCAAGGGCATGAGCTAC
**pMal-c2X-DV3-R**	TGCCTGCAGGTCGACTCTAGATTATTAGCCTTTACGGTACCA
**pMal-c2X-DV4-F**	GCAGACTAATTCGAGCTCCAAAGGTATGAGCTAC
**pMal-c2X-DV4-R**	TGCCTGCAGGTCGACTCTAGATTATTAACCTTTACGGAACCA
**Mutagenesis**	
**HCDR2-A54E-F**	GATCCGGAAAACGGTTATTCTAAATATGACCCGAA
**HCDR2-A54E-R**	ACCGTTTTCCGGATCGATACGACCAATCCATTCCA
**HCDR3-Y105R-F**	GGTTTCGCGCGTTGGGGTCAGGGTACCCTGGTTAC
**HCDR3-Y105R-R**	CTGACCCCAACGCGCGAAACCTTCGTAATCACGCG
**Sequencing**	
**pComb3XSS-light-chain-F**	AAGACAGCTATCGCGATTGCAG
**pComb3XSS-light-chain-R**	CGGCTGCCGTAGGCAATAGGT
**pComb3XSS-heavy-chain-F**	ACCTATTGCCTACGGCAGCCG
**pComb3XSS-heavy-chain-R**	AGAAGCGTAGTCCGGAACGTC
